# Main Determinants of Catastrophic Health Expenditures: A Bayesian Logit Approach on Iranian Household Survey Data (2010)

**DOI:** 10.5539/gjhs.v7n4p335

**Published:** 2015-01-25

**Authors:** Ali Akbar Fazaeli, Hossein Ghaderi, Amir Abbas Fazaeli, Farhad Lotfi, Masoud Salehi, Mohsen Mehrara

**Affiliations:** 1 Departments of Health Economics, School of Health Management and Information Sciences, Iran University of Medical Sciences, Tehran, Iran; 2 Health Management and Economics Research Center, Iran University of Medical Sciences, Tehran, Iran; 3 Department of Statistics and Mathematics, School of Health Management and Information Sciences, Iran University of Medical Sciences, Tehran, Iran; 4 School of Economics, Tehran University, Tehran, Iran

**Keywords:** catastrophic health expenditures, household survey data, health equity, Bayesian logit model

## Abstract

**Background:**

During recent decades, increase in both health care expenditures and improvement of the awareness as well as health expectations have created some problems with regard to finance healthcare expenditures so that the issue of health financing by households has been determined as a major challenge in health sector. According to the definition by the World Health Organization, catastrophic health expenditure is considered if financial contribution for health service is more than 40% of income remaining after subsistence needs have been met.

**Objectives:**

The purpose of our study was determination of Main factors on catastrophic health expenditures in Iranian households.

**Patients and Methods:**

In this study, using an econometrics Bayesian logit model, determinants of the appearance of catastrophic health expenditure based on household budget data collected in 2010 were evaluated.

**Results:**

Among Iranian households, the following groups were more likely to encounter with unsustainable health expenditures: rural households, households with the numbers of the elderly more than 65 years, illiterate householders, unemployed householders, households with some unemployed persons, households in upper rank and households with larger equivalent household size were higher than the average of community could significantly predict catastrophic health expenditures.

**Conclusions:**

About 2.1% of households were faced with catastrophic health expenditures in 2010. Thus, the implemented policies could not make considerable and significant change in improving justice in financing in health systems.

## 1. Introduction

The World Health Organization (WHO) introduced fair financial contribution for health funding as one of the three main objectives in health system (WHO, 2001). Health care usually is a luxury good (Mehrara & Fazaeli, 2009). Within the recent decades, increased health care expenditures due to technology development as well as increased awareness and expectations of health have created some problems with regard to finance healthcare expenditures. Some results of unfair conditions in the health sector include (Wagstaff, 2007; Mehrolhassani, 2014): 1) Vulnerable households suffer from great difficulties in providing financial requirements of health services and thus decrease some other necessary expenses that result in a decline in household welfare conditions. 2) High financial load of healthcare systems due to lower savings and lower income allocated to other requirements, especially for food or education of children as national capitals may be resulted in reduced household productivity as a key factor in the national production processes. Therefore, negative effects of the failure of the financial system for the health sector on production and economic growth of the society can be observable. 3) Some low-income households around minimum poverty line lose their livelihood ability and income and assets because of the compulsory providing medical expenses and therefore face with catastrophic health expenditures and finally experience indigence and impoverishment (Rahman, 2013). According to the definition by the World Health Organization, catastrophic health expenditure is considered if financial contribution for health service is more than 40% of income remaining after subsistence needs have been met (Ke Xu et al., 2007; Yardim, Cilingiroglu, & Yardim, 2010). Iran’s fourth five year developmental plan act in its 90th article, articulated decreasing household’s exposure to catastrophic health expenditure to one percent. We aimed to assess degree of success in achieving the mentioned objective at the end of forth five year developmental plan (M. Mehrara & Fazaeli, 2010; H. G. P. Student & H. S. B. P. Student, 2014). In the present study, using an econometrics Bayesian logit model, determinants of the appearance of catastrophic health expenditure in households based on household budget data collected in 2010 were evaluated.

Xu et al. using a cross-country analysis design collected their data from household surveys to explore, by regression model, variables associated with health expenditure. They defined expenditure as being catastrophic if financial contribution to the health services is more than 40% of income remaining after subsistence needs have been met. They finally found that people can be protected from catastrophic expenditures by reducing a health system’s dependency on out-of-pocket payments. In their analysis, three main preconditions for catastrophic expenditures were the availability of health services requiring payment and the lack of prepayment or health insurance (K. Xu et al., 2003).

Ekman purpose of their study by in a low-income country was to quantity analyze the role of health care insurance in the key determinants of catastrophic expenditures. He showed that the health care insurance didn’t provide financial protection against catastrophic expenditures risk, even insurance increased the risk (Ekman, 2007).

Nekoei Moghadam et al. indicated 2.8% of households were faced to catastrophic health expenditures. The main factors of catastrophic healthcare were ambulatory utilization, hospitalization services, pharmaceutical addiction cessation services, and pharmaceuticals consumption. Iranian health sector has not reached the objective of decreasing catastrophic expenditure to one percent. Inefficient medical insurance coverage, different fee plans practiced by public and private providers, inefficient referral system are assumed as probable obstacles toward reducing households’ facing to catastrophic expenditures (Moghadam, Banshi, Javar, Amiresmaili, & Ganjavi, 2012).

Hajizadeh and Nghiem using ordered-probit model indicated length of stay, lower wealth index of households, and private hospital care utilization are major determinants contributing to increase in the probability of catastrophic medical expenditures. Also, they found living in Sistan and Balochestan, East Azarbaijan and Kordestan lead to higher level of catastrophic health expenditures in Iran. Based on their results, the current employer-employee health insurance didn’t offer equal protection against hospital expenditures. It appears that a single universal health care insurance plan that covers services for all individuals regardless their employment situation can better protect them from catastrophic health payment (Hajizadeh & Nghiem, 2011).

Kavosi et al. found that the proportion of households facing catastrophic expenditures had no significant change in the period (12.6% in 2003 vs 11.8% in 2008). The key factors of catastrophic expenditures for both years were medical utilization and health insurance status. Meanwhile the status of socio-economic was the principle contributor to inequality in catastrophic payments, Also, unequal utilization of outpatient and dentistry services had decreased the inequality in catastrophic expenditures among socio-economic categories (Kavosi et al., 2012).

### 1.1 Objectives

The purpose of our study was determination of Main factors on catastrophic health expenditures in Iranian households in 2010.

## 2. Patients and Methods

This study was done based on a methodology introduced by the World Health Organization and using field data plan on expenditures and income statistics that are collected each year by the Iran Statistical Center. The total number of households in 1386 was over 28,997 households out of the total of 12,311 households were living in rural regions and 16,686 households residing in urban regions. In this study, the term of “Out Of Pocket” or (OOP) and “Capacity to pay” or (CTP) were defined as the paying out of pocket and payment capacity or capability respectively. CTP of the household was considered as total revenue in excess of expenses of the minimum wage. The rise in this index means that households are forced to pay for their excess capacity to preserve the expenditure of family health. Therefore, a critical limit has been defined that exceed this critical level is considered as catastrophic health expenditures. Health economists and international references have defined this crisis level as 40% based on various studies and experiences. Careful analysis of the catastrophic state of health expenditures without identification of targeted households is impossible. Thus, it is necessary to identify households with greater likelihood of catastrophic health expenditures on the basis of their socioeconomic parameters. Among common usable statistical and econometric models, this study applied Bayesian logit model for assessing effects of socioeconomic variables on the probability of catastrophic expenditures. In these models, dependant variables scored as 1 (for households with catastrophic expenditures) or 0 (for other households). This dependant item (Cata_h_) is categorized as (Wagstaff, 2007).


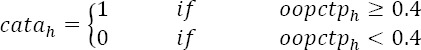


So that oopctp_h_ is the pay out of pocket payments of households to payment capacity of that household. Independent variables include:

**Ur** (scores 1 for rural households and score 0 for urban households);

**UP65_Num** (the number of individuals older than 65 years in each household);

**BELOW 5_Num** (the number of individuals younger than 5 years);

**H_Sex** (scores 0 for men and score 1 for women);

**H_Education** (score 1 for illiterate or low literacy householder and score 0 for others);

**H_Employ** (score 1 for employed householder and score 0 for unemployed ones);

**Employ_num** (the number of employed persons in household);

**Insurance** (score 1 for insured household and score 0 for uninsured household)

**Eq_size** (presents equivalent household size – household expenditures increases nonlinearly by increase of the number of household members with) 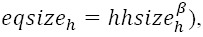 *β*=0.56, h= household);

**H_Married** (1 score for married and score 0 for single ones);

**Rank** (Number of household expenditure deciles);

In Table 1, we showed total variables and their explanations used in the model. The advantage of this study over previous studies was that an econometric model with a wide set of explanatory variables (quantitative and qualitative) was used for analyzing and estimating marginal impacts of these variables. Also in this study we use from Bayesian analysis with R software to achieve exact output.

**Table 1:** Total variables and their explanations used in the Model

Repressor	Explain
Employ _Num	the number of employed persons in household
Insurance	1 for insured household and score 0 for uninsured household
Rank	Number of household expenditure deciles
H _Sex	score 0 for men and score 1 for women
Up65_Num	the number of individuals older than 65 years in each household
Ur	score 1 for rural households and score 0 for urban households
H _Education	score 1 for illiterate or low literacy householder and score 0 for others
Below5_Num	the number of individuals younger than 5 years
H _Married	1 score for married and score 0 for single ones
H _Employ	score 1 for employed householder and score 0 for unemployed ones
Eq_size	presents equivalent household size
**Depended variable**	**Explain**
Catastrophic	catastrophic health expenditures=1& the other=0

## 3. Results

**Figure 1:**
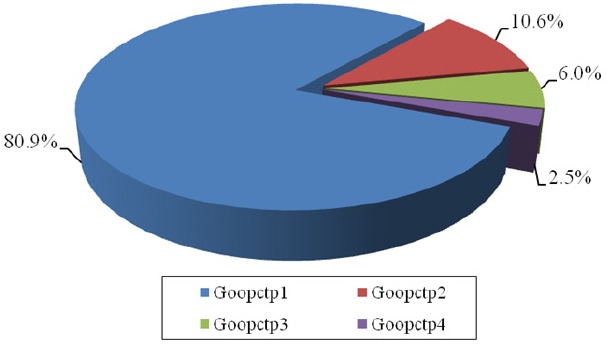
The proportion of the population to break the household budget share of health care expenditures in 2010

**Figure 2:**
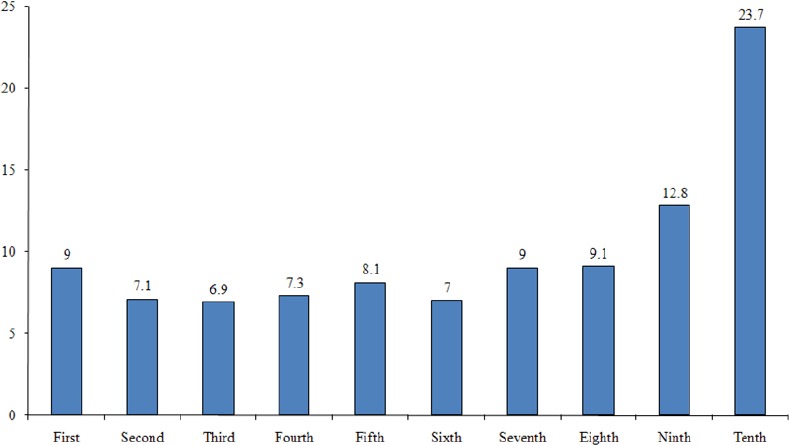
Distribution of households with catastrophic health expenditures in different expenditure percentiles in 2010


According to Figure 1, the proportion of households in group Goopctp4 was 2.5%. On the other hand, 2.5% of people experienced catastrophic health expenditures. Also, the proportion of households in group Goopctp 3 was 6%, in group Goopctp 2 was 11%, and in Goopctp 1 was 80.5%, respectively. Figure 2 displays the distribution of households that faced to catastrophic health expenditures between the budget groups in Iranian households 2010. In Figure 1, the proportion of the population to break the household budget share of health care expenditures, after deducting the cost of living in each group is shown as follows: Goopctp 1) is the proportion of households that paid 0% to 10 % of their capacity to for health expenditures; Goopctp 2) is the proportion of households that paid 10% to 30% of their capacity to for health expenditures; Goopctp 3) is the proportion of households that specified 30% to 40% of their capacity to for health expenditures; Goopctp 4) is the proportion of households who faced to catastrophic health expenditures. We estimate the following model:

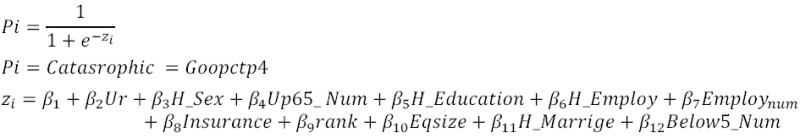


and for examine marginal effect (Maddala, 1983):


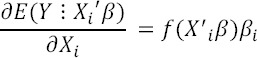


Bayesian models more flexible & handles complex models. Bayesian logistic analyses follows pattern for all Bayesian analyses: Likelihood function, Form a prior distribution & use Bayes theorem to estimate the posterior distribution. In Table 2 we show Mean, standard deviation, marginal effect and quintiles in posterior distributions.

**Table 2:** Mean, standard deviation, marginal effect and quintiles in posterior distributions

	Mean	SD	2.5%	50%	97.5%	Marginal effect
**(Intercept)**	-4.5098	0.2150	-4.9307	-4.4958	-4.0726	
**Employ _Num**	-0.2439	0.0644	-0.3749	-0.2391	-0.1299	-0.0057
**Insurance**	0.0669	0.0862	-0.1159	0.0632	0.2415	0.0014
**Rank**	0.1552	0.0541	0.0573	0.1532	0.2722	0.0037
**H_ Sex**	-0.0906	0.1244	-0.3271	-0.0950	0.1440	-0.0021
**Up65_Num**	0.4794	0.0617	0.3569	0.4786	0.6112	0.0113
**Ur**	0.9156	0.0894	0.7499	0.9212	1.0854	0.0219
**H _Education**	-0.4568	0.0937	-0.6621	-0.4514	-0.2657	-0.0108
**Below5_Num**	0.0054	0.0832	-0.1766	0.0084	0.1672	0.00009
**H_ Married**	0.0702	0.0482	-0.0886	0.0474	0.1765	0.0010
**H_ Employ**	-0.2595	0.1082	-0.4553	-0.2632	-0.0473	-0.0064
**Eq _ size**	0.2051	0.0960	0.0170	0.1976	0.4024	0.0045

## 4. Conclusion

The implemented policies could not considerable and significant change in improving justice in financing in health systems. According to the study findings, 2.1% of people experienced catastrophic health expenditures. Also, the proportion of households in Goopctp3 (the proportion of households that paid 10% to 30% of their capacity to for health expenditures) was 6%, in Goopctp2 (the proportion of households that paid 30% to 40% of their capacity to for health expenditures) was 11%, and in Goopctp1 (the proportion of households that specified less than 10% of their capacity to for health expenditures) was 80.5%, respectively. In addition, predicting determinants of the appearance of catastrophic expenditures using Bayesian logit model showed that among all variables, rural households (21% marginal effect), households which the numbers of the elderly more than 65 years (11% marginal effect), households with illiterate head (10.8% marginal effect), unemployed householders (6.4% marginal effect), households with the number of unemployed persons in household (5.7% marginal effect), households in upper rank and households with larger equivalent household size were higher than the average of community could significantly predict catastrophic health expenditures. In this regard, health insurance do not has significant effect on catastrophic health expenditure.
